# Genetic Diversity, Population Structure and Linkage Disequilibrium Assessment among International Sunflower Breeding Collections

**DOI:** 10.3390/genes11030283

**Published:** 2020-03-06

**Authors:** Carla V. Filippi, Gabriela A. Merino, Juan F. Montecchia, Natalia C. Aguirre, Máximo Rivarola, Guy Naamati, Mónica I. Fass, Daniel Álvarez, Julio Di Rienzo, Ruth A. Heinz, Bruno Contreras Moreira, Verónica V. Lia, Norma B. Paniego

**Affiliations:** 1 Instituto de Agrobiotecnología y Biología Molecular–IABiMo–INTA-CONICET, Instituto de Biotecnología, Centro de Investigaciones en Ciencias Veterinarias y Agronómicas, Instituto Nacional de Tecnología Agropecuaria, Hurlingham 1686, Argentina; 2 Programa Académico para la Investigación e Innovación en Biotecnología, Universidad Nacional de Moreno–UNM, Moreno 1744, Argentina; 3 European Molecular Biology Laboratory, European Bioinformatics Institute, Wellcome Genome Campus, Hinxton, Cambridge CB10 1SD, UK; 4 Instituto de Investigación y Desarrollo en Bioingeniería y Bioinformática–IBB, Consejo Nacional de Investigaciones Científicas y Técnicas (CONICET), Universidad Nacional de Entre Ríos, Oro Verde 3100, Argentina; 5 Instituto de Investigación en Señales, Sistemas e Inteligencia Computacional-sinc(i), Consejo Nacional de Investigaciones Científicas y Técnicas (CONICET), Universidad Nacional del Litoral, Santa Fe 3000, Argentina; 6 Estación Experimental Agropecuaria INTA Manfredi, Manfredi 5988, Argentina; 7 Facultad de Ciencias Agropecuarias, Universidad Nacional de Córdoba, Córdoba 5000, Argentina

**Keywords:** sunflower, breeding, linkage disequilibrium, population structure, genetic diversity

## Abstract

Sunflower germplasm collections are valuable resources for broadening the genetic base of commercial hybrids and ameliorate the risk of climate events. Nowadays, the most studied worldwide sunflower pre-breeding collections belong to INTA (Argentina), INRA (France), and USDA-UBC (United States of America–Canada). In this work, we assess the amount and distribution of genetic diversity (GD) available within and between these collections to estimate the distribution pattern of global diversity. A mixed genotyping strategy was implemented, by combining proprietary genotyping-by-sequencing data with public whole-genome-sequencing data, to generate an integrative 11,834-common single nucleotide polymorphism matrix including the three breeding collections. In general, the GD estimates obtained were moderate. An analysis of molecular variance provided evidence of population structure between breeding collections. However, the optimal number of subpopulations, studied via discriminant analysis of principal components (K = 12), the bayesian STRUCTURE algorithm (K = 6) and distance-based methods (K = 9) remains unclear, since no single unifying characteristic is apparent for any of the inferred groups. Different overall patterns of linkage disequilibrium (LD) were observed across chromosomes, with Chr10, Chr17, Chr5, and Chr2 showing the highest LD. This work represents the largest and most comprehensive inter-breeding collection analysis of genomic diversity for cultivated sunflower conducted to date.

## 1. Introduction

Sunflower (*Helianthus annuus spp. macrocarpus*) is one of the most important oilseed crops, with a global production value estimated at USD 20 billion per year (FAO 2016). Its early domestication occurred in the interior mid-latitudes of eastern North America ca. 4000 years ago, but it became an oil crop only when it reached Russia late in the XVIIIth century. The foundational efforts of Pustovoit at VNIIMK to develop high yielding, open-pollinated varieties with high oil content are considered the main genetic base of modern sunflower breeding [1]. After that, the discovery of cytoplasmic male sterility at the Institut National de la Recherche Agronomique (INRA, France) in a cross between *Helianthus petiolaris* and cultivated sunflower [2] and fertility restoration genes [3] at the United States Department of Agriculture (USDA, USA) was fundamental to allow sunflower hybrid production.

Argentina has a long tradition in sunflower breeding. Since 1931, and by exploiting the diversity of a broad range of foreign genetic resources in combination with introgressions of wild *Helianthus* species (e.g., *H. annuus, H. argophyllus,* and *H. debilis* ssp. *cucumerifolius*), the Instituto Nacional de Tecnología Agropecuaria (INTA) has pioneered sunflower breeding and has become one of the most prolific sunflower breeders in the country [4,5,6].

Today, the major sunflower producing countries are Ukraine, Russia, the European Union, and Argentina. According to Vear [1], in spite of the fact that Ukraine and Russia produce almost half of the world’s sunflower seeds, the main research and breeding programs are concentrated in western Europe, USA–Canada, and Argentina. In these countries, especially the United States Department of Agriculture (USDA), the University of British Columbia (UBC) in Canada, INRA in France, and INTA in Argentina, public research provides the greater part of the breeding efforts, together with basic science.

Over the last years, these institutions made a significant contribution towards the development and characterization of proprietary breeding and pre-breeding collections [4,5,7,8,9,10,11,12,13,14]. However, a comprehensive analysis of the genetic diversity and allelic variants currently being used across international breeding programs has not yet been undertaken. Conducting such studies is an essential step to understanding the genetic base of current sunflower breeding worldwide. This knowledge can help with the decision-making process during the incorporation of new genetic backgrounds and/or the mining of gene banks and crop wild relatives [15].

With the publication of the first sunflower reference genome [12], a large amount of genomic data became available at public repositories, including breeding and pre-breeding materials from INRA and USDA-UBC (low coverage whole genome resequencing data [12,13]), thus allowing the unequivocal comparison of genetic data from different sources.

In this work, we implement for the first time a double digest RAD seq approach [16] to genotype a panel of 135 sunflower inbred lines belonging to the INTA breeding program. By combining proprietary data with data coming from public next-generation sequencing repositories, we persued the following goals: (a) to assess the distribution of genetic diversity within and between breeding programs; (b) to identify and characterize worldwide patterns of population structure in cultivated sunflower; and (c) to estimate the extent of linkage disequilibrium (LD) in the different germplasm groups. Our results provide reliable estimates of the variability levels within sunflower collections worldwide and allow the determination of the distribution pattern of global diversity.

## 2. Materials and Methods

### 2.1. Genotyping

#### 2.1.1. INTA Collection

Data generation: The pre-breeding collection of INTA, composed of 135 sunflower inbred lines preserved at the Active Germplasm Bank of INTA Manfredi (AGB-IM), was genotyped using a double digest restriction-site associated DNA sequencing (ddRADseq) protocol adapted from Peterson et al. [16]. Two rare cutter restriction enzymes -SphI and EcoRI-, one of them methylation-sensitive, were used to produce the DNA libraries. Fragments were manually selected on agarose gels after adapter-ligation at sizes ranging from 340 to 550 bp, corresponding to 260–470 bp of original genomic DNA fragments. The ddRADseq protocol and paired-end 2 × 125 bp sequencing (Illumina, HiSeq 2500 platform, San Diego, CA, USA) were carried out at the Istituto di Genomica Applicata (IGA, Udine, Italia). The 135 sunflower inbred lines are listed in Table S1.

Variant calling: Raw Illumina reads were de-multiplexed and quality-checked using the *process_radtags* routine implemented in Stacks (v1.42 [17]). After the removal of variable-length barcode sequences, all reads were trimmed to 110 bp. Bowtie2 aligner with default parameters [18] was used to align the reads to the reference genome (XRQ inbred line, GCA_002127325.1, [12], retrieved from plants.ensembl.org, [19]). Single nucleotide polymorphisms (SNPs) were called using the *ref_map* routine implemented in Stacks software [17], as described in Aguirre et al. [20]. Additional cleaning of ambiguous alleles and/or putative sequencing errors was carried out with the *rxstacks* module by removing SNP calls with the likelihood below -10 and accepting loci with a maximum of 50% sample carrying confounding alleles (i.e., excess of alleles or matching more than one catalog locus). Only biallelic positions were kept for further analysis. Finally, the *Populations* command was used to generate the final VCF file.

ddRADseq data exploration and VCF filtering: Filters related to the percentage of missing data (80%), number of SNPs per sequenced region or tag (no more than 4 SNPs/tag), and minor allele frequency (MAF >0.05) were applied to the VCF matrix using R and custom scripts. Plots were performed using the R package “ggplot2” [21].

#### 2.1.2. INRA and USDA-UBC Collections

Circa 10 TB of low-coverage (~10×) whole genome sequencing (WGS) data were retrieved from the European Nucleotide Archive [22]. This corresponds to a total of 545 fastq files (project PRJNA353001: 464 fastq files corresponding to 289 USDA-UBC sunflower accessions and project SRP092899: 81 fastq files corresponding to 58 INRA sunflower accessions). FastQC [23] was employed for visual inspection of the sequence quality, and Trimmomatic [24] was used for Illumina TruSeq adaptor trimming and quality filtering. After that, raw reads were aligned to the reference genome using Bowtie2 aligner with default parameters [18]. For each sample, variants were called using GATK UnifiedGenotyper [25] with the “GENOTYPE_GIVEN_ALLELES” option, giving as input the VCF file obtained for the INTA collection, in order to obtain the same panel of SNPs for the three pre-breeding collections (i.e., INTA, INRA, and USDA-UBC). The GATK parameters “min_base_quality_score” and “stand_call_conf” were set to 30 in order to discard low confident SNPs. The command line used for SNP calling from WGS data is available in Supplementary File S1.

### 2.2. Missing Data Imputation

For each of the three VCF matrices, we used the imputation strategy proposed by Merino [26], which exploits the SNPs correlation structure and uses it for genotype prediction through Random Forests. The imputation source code is freely accessible in the SNPsRFImputation repository (https://github.com/gamerino/SNPsRFImputation). After imputation, given that the methodology discards those SNPs that cannot be accurately imputed, an intersection of the three matrices was done in order to obtain the same set of SNPs for all populations. The variants called in this work for the three sunflower populations have been submitted to Ensembl Plants (plants.ensembl.org, [19]), where they can be displayed interactively and downloaded in bulk.

### 2.3. SNP Variant Characterization

The Variant Effect Prediction (VEP) tool [27], available at plants.ensembl.org [19], was used to predict the potential effect of each genotyped variant. Variant consequences and impact percentages were plotted and used for variant characterization.

### 2.4. Genetic Diversity Analysis

Measures of genetic diversity, including unbiased expected heterozygosity (He), observed heterozygosity (Ho), allele frequency and minor allele frequency (MAF) were estimated between and within populations, using the R packages “PopPR” [28] and “Adegenet” [29]. Allele frequency plots were generated using “Adegenet” [29].

### 2.5. Population Structure Analysis

The extent of differentiation between INTA, INRA, and USDA-UBC was investigated via analysis of molecular variance (AMOVA), using the R Package “PopPR” [28]. Statistical significance was evaluated by doing 999 permutations.

The Bayesian approach implemented in STRUCTURE [30,31] was used to infer population structure for the whole panel of accessions. The number of clusters evaluated ranged from 1 to 20 with 4 runs per K value. For each run, the initial burn-in period was set to 100,000 with 100,000 MCMC iterations. To determine the most probable value of K, the deltaK method described by Evanno et al. [32] was used as implemented in Structure Harvester [33]. Accessions were assigned to a given population when the inferred ancestry was >0.70.

Genetic relationships among accession were also examined by applying discriminant analysis of principal components (DAPC, [29]). The function DAPC was executed using the clusters identified by K-means [34]. The number of clusters was assessed using the function ‘*find.clusters*’, evaluating a range from 1 to 40. The optimal number of clusters was chosen on the basis of the lowest associated Bayesian information criterion (BIC).

In addition, a relatedness analysis using Identity-By-Descent (IBD) measures between all the accessions was performed using the R package “SNPRelate” [35].

In order to compare with previous work [5,8,9,11], and when information was available, accessions were classified as belonging to one of the two major heterotic groups (RHA—restorer lines and HA—maintainer lines, Table S1). A principal component analysis (PCA) was done using the basic R “prcomp” function, and the first two principal components were graphed in a two-dimensional space. Accessions were colored according to their maintainer/restorer status.

A total of ten (10) public sunflower inbred lines that were present in more than one breeding collection were compared using the percentage of shared alleles to assess potential discrepancies among collections.

### 2.6. Linkage Disequilibrium

The extent of LD was estimated using the R package “Synbreed” [36] with a gateway to the PLINK software [37], which estimates pairwise LD between markers. Measures of r^2^ vs. physical distance (bp) per chromosome were plotted using the R package “ggplot2” [21]. The y ~log(x) function was applied in order to fit the extent of r2 decay. A heatmap of pairwise LD between markers was plotted using the basic R “heatmap” function.

## 3. Results

### 3.1. ddRADseq in INTA Accessions

**Figure 1:**
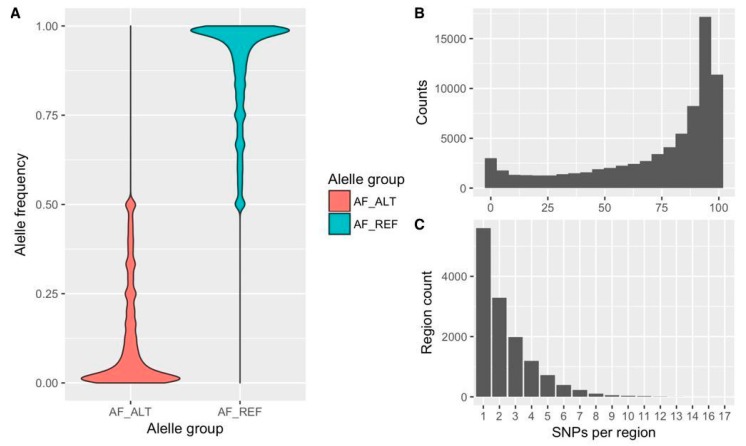
Initial characterization of the INTA ddRADseq matrix before filtering. (**A**) Allele frequency. AF_ALT, alternative allele (i.e., less frequent allele); AF_REF, reference allele (i.e., most frequent allele); (**B**) Percentage of missing data (*x*-axis: percentage); (**C**) Number of SNPs per tag, or sequenced region (110 bp).

A total of 126 M reads were produced across six pooled batches with 24 inline barcodes along with a 6-bases TruSeq indexing system to tag each pool. After de-multiplexing, about 125 M reads were available for downstream analyses. Along with the removal of barcode sequences, reads were all clipped to a fixed length of 110 bp in order to (i) remove low-quality bases at the 3′-ends and (ii) maintain a consistent length given the variable length of the barcodes. This processing was necessary to prevent any incompatibility issues with downstream analysis software. An average of 930,000 reads per sample was generated (ranging from 217,923 to 1,497,629), and ~97% of them mapped against the reference genome [12]. The variant calling algorithm implemented in Stacks yielded a total of 155,390 SNPs genotyped in at least one sample. From this, 76,094 had MAF <0.05 and were discarded (Figure 1A). Moreover, 43,398 SNPs were eliminated because of high levels of missing data (>80%, Figure 1B). In addition, 1582 variants called in tags that had more than four SNPs were also discarded (i.e., more than 4 SNPs/110 bp, Figure 1C). Finally, 7 SNPs that mapped against the plastome were removed, yielding a final matrix of 34,309 SNPs genotyped in the INTA accessions. The percentage of each substitution type was examined in the final matrix, with transitions being the most frequent genetic change (Figure S1).

This final matrix was used as input for variant calling under the “GENOTYPE_GIVEN_ALLELES” option in GATK for the USDA-UBC and INRA data.

### 3.2. Variant Analysis of INRA and USDA-UBC Accessions

From the low-coverage (~10x) WGS data retrieved from ENA, an average of 1.25% (ranging from 0% to 5.99%) of the USDA-UBC reads and 2.47% (ranging from 0.29% to 7.53%) of the INRA reads were discarded after trimming. Of these, 96.69% (68.60–98.44%) of USDA-UBC data and 97.27% (94.53–99.38%) of INRA data mapped against the reference genome. From the initial 34,488 SNP list used as input for variant calling, 22,207 SNPs in the USDA-UBC population and 20,481 SNPs in the INRA population passed the filters specified for variant calling and were genotyped in at least one accession.

### 3.3. Missing Data Imputation

The imputation algorithm uses correlation and LD to select the predictors from the list of SNPs fully genotyped in each population. For the INTA data, a total of 1697 SNPs were genotyped in all accessions, while 842 and 7789 were fully genotyped in the USDA-UBC and INRA breeding collections, respectively. After imputation, a total of 20,750, 18,525, and 18,925 SNPs were obtained for INTA, USDA-UBC and INRA populations, respectively.

**Figure 2:**
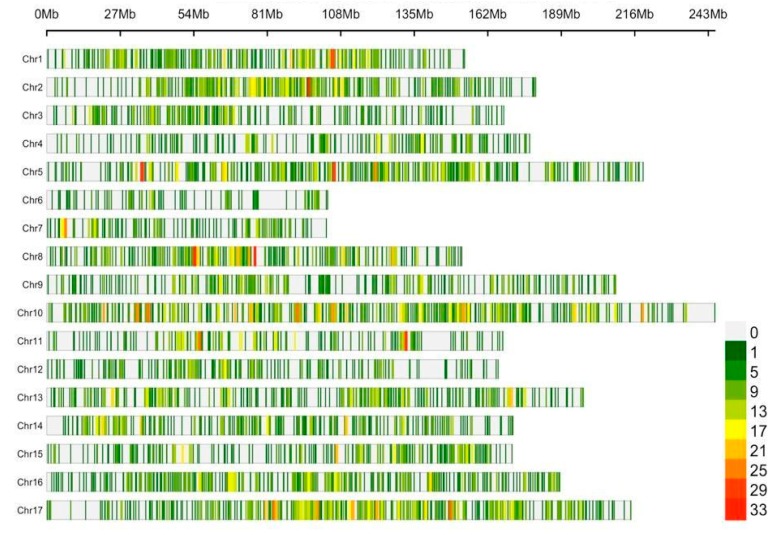
Distribution of SNPs along the 17 sunflower chromosomes. The colors indicate the number of markers within a 1 Mbp window.

Intersecting the imputed matrices rendered a total of 11,834 SNPs in common between the three breeding collections. The markers showed a uniform distribution in all the sunflower chromosomes (Figure 2), varying from 194 in Chr6 to 1330 in Chr10, being the number of SNPs in accordance with chromosome length.

### 3.4. SNP Effect Prediction

**Figure 3:**
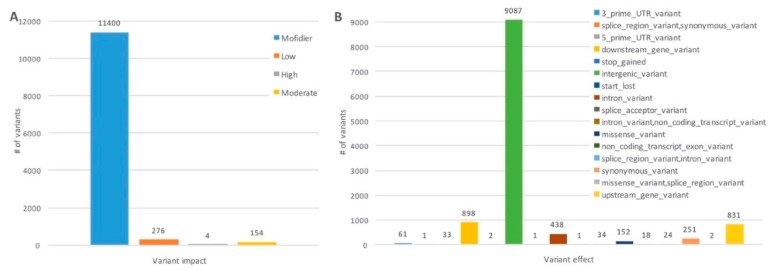
Variant effect predictor output for the 11,834 SNPs used in subsequent analysis. (**A**) Variant impact. (**B**) Variant consequences, according to genome site location.

The predicted consequences of all the genotyped SNPs, classified in 16 terms defined by the Sequence Ontology [38], as well as their impact rating (i.e., potential impact in protein behavior) are shown in Figure 3A,B. Most of the variants were predicted as intronic, intergenic or located at 5′ or 3′ regions of a gene, with only 1.33% of the polymorphism being classified as moderate or high impact variants.

### 3.5. Genetic Diversity (GD) Analysis

The GD values expected heterozygosity (He), observed heterozygosity (Ho) and minor allele frequency (MAF) were estimated for the full panel of accessions, as well as for each breeding collection. The results are presented in Table 1.

Regardless of the population size (n), the He and Ho values were comparable between breeding collections. A total of 18 SNPs (located in Chr2, positions 49845689, 54744211, 54744259, 60811569, 63947124, 85115362, 88670408, 90061552, 93707569, 99976375, 99976502, 100213237, 102712361; Chr5 pos. 156213971; Chr15 pos. 37616622, 37616623; and Chr17 pos. 69089493 bp) had low frequency (MAF <0.05) in both INRA and USDA-UBC collections, but had MAF >0.05 in INTA accessions. A graphical representation of individual heterozygosis between and within populations is presented in Figure S2. The uniformity and absence of polymorphisms with respect to the reference genome observed in the accessions located in the upper part of Figure S2B (INRA), is due to the fact that those accessions correspond to three independent replicates of the inbred line XRQ, the same used to generate the sunflower reference genome [12].

### 3.6. Population Structure Analysis

The analysis of molecular variance (AMOVA) using 11,834 SNPs revealed significant genetic differentiation of populations, with variation among breeding collections, representing 4.58% of the total genetic variance (*p* < 0.01, 999 permutations).

**Figure 4:**
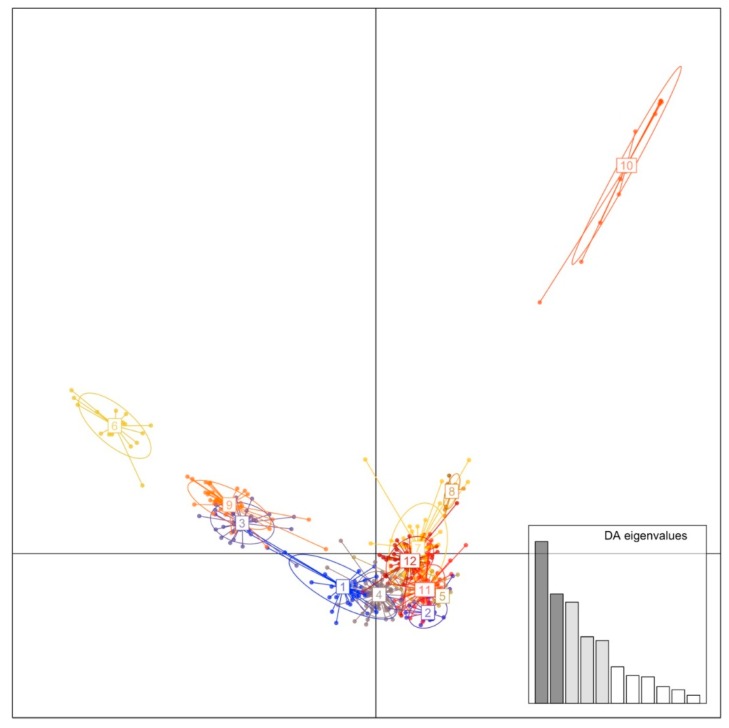
Scatter plot of DAPC showing the first two principal components for K = 12. Dots represent accessions while the ellipses represent the 12 groups. Eigenvalues of the analysis are also displayed.

Bayesian population structure analysis for the whole panel of accessions, including INTA, INRA, and USDA-UBC retrieved a maximum deltaK at K = 6 (Figure S3) with a second maximum at K = 4. Inspection of the DAPC plot also revealed the presence of genetic structure among these accessions. The sequential k-means algorithm identified 12 groups, and the eigenvalues of the analysis showed that most of the genetic structure was captured by the first five PCs (Table S1, Figure 4). Although the AMOVA provided evidence of population structure between breeding collections, the optimal number of subpopulations is difficult to determine since no single unifying characteristic is apparent for any of the inferred groups, independently of the clustering method.

In spite of the lack of clear associations between accession origin (i.e., breeding program) and the 6 groups retrieved from STRUCTURE or the 12 groups retrieved from DAPC, some clusters were consistently enriched with accessions from a single origin (Table S2A,B). According to STRUCTURE assignments, Group 3 is composed of USDA-UBC accessions, Group 4 is composed mainly of INTA accessions, while the remaining groups are of mixed origin. The DAPC plot based on the first two PCs showed that Groups 6 and 10, composed only by USDA-UBC accessions, are the most differentiated (Figure 4). In addition, DAPC Group 1 is composed mainly of INTA accessions, while the remaining groups show a mixture of origins.

Distance matrices based on IBD were constructed for all pairs of individuals. Distances varied from 0.000 to 0.389, with an average of 0.172. The function cut-tree identified 9 groups (Table S2C). The dendrogram depicting the relationships among sunflower accessions is provided in Figure S4. The correspondence in the IBD group assignment with DAPC and STRUCTURE is presented in Table S3A,B.

A total of 389 out of 482 accessions were classified as HA/RHA, while the remaining 93 accessions for which no information was available, were kept as N/A. The distinction between HA and RHA was displayed on the second axis of the PCA, although an overlapping zone can be seen (Figure S5). The first two principal components (PCs) captured a low percentage of the variance (7.84% and 6.98%, respectively).

### 3.7. Comparison of Duplicated Samples between Breeding Collections

The list of duplicated inbred lines, along with their inter-collection identity estimates, is presented in Table 2. Eight of these comparisons showed a proportion of shared alleles above 92%, while the remaining two (i.e., HAR2 and RHA299) exhibited lower values (Table 2). Independently of the clustering method, those duplicated accessions showing less than 8% differences clustered together (Figure S4, Table S1). Inbred lines that were present in more than one collection and clustered together were underscored in the dendrogram representation or highlighted in Figure S1.

### 3.8. Linkage Disequilibrium

The patterns of LD decay, measured as r^2^, were obtained for the full panel of accessions, as well as for each of the breeding collections. More than 4.8 M pairwise comparisons were done per breeding population.

**Figure 5:**
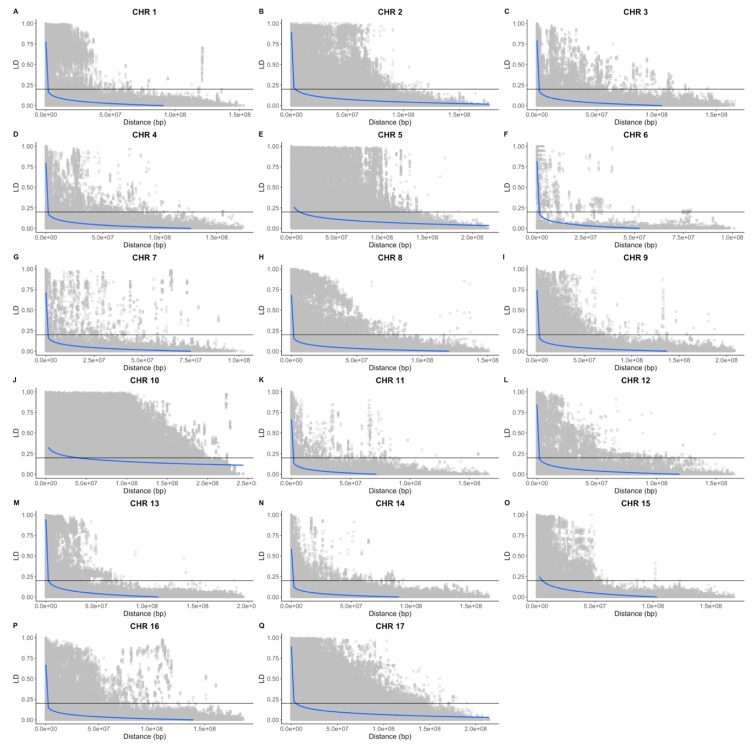
Linkage disequilibrium (r^2^) vs. physical distance (bp) for the full panel of accessions. A cut-off line was plotted at r^2^ = 0.2. The blue line represents the *y* ~log(x) function. (**A**–**Q**) Sunflower chromosomes 1 to 17. CHR = chromosome

The results of the LD analysis are summarized in Figure 5A–Q, Figures S6A–Q, S7A–Q, S8A–Q and Table S4. A heatmap of pairwise LD per chromosome for the full panel of accessions is depicted in Figure S9. Different overall patterns of LD are apparent when looking across chromosomes, but in general, these patterns are consistent whether the full panel of accessions or the individual breeding collections are considered. Chr10 exhibits the highest LD (mean r^2^ ranging from 0.19 to 0.22), independently of the set of accessions under analysis, followed by Chr17 (mean r^2^ ranging from 0.11 to 0.16), Chr5 (mean r^2^ ranging from 0.12 to 0.15), and Chr2 (mean r^2^ ranging from 0.11 to 0.14). The INRA breeding population also showed high LD at Chr15 (mean r^2^: 0.11). On the other hand, Chr14 showed the lowest LD (mean r^2^ ranging from 0.04 to 0.05). Visual inspection of the heatmap showed specific non-recombining regions (i.e., Ch15) and chromosomes with reduced recombination (e.g., Chr 10).

## 4. Discussion

Germplasm collections are valuable resources for crop improvement. However, to fully unlock their potential, it is critical to have detailed information about the amount and the distribution of the genetic diversity available within collections. Through the integration of genomic data for three of the most studied sunflower breeding collections, INTA, INRA and USDA-UBC, this work represents the largest and most comprehensive analysis of genetic diversity, population structure and linkage disequilibrium for cultivated sunflower conducted to date.

Reduced representation sequencing methods, as genotyping by sequencing (GBS) and double digest RAD seq (ddRADseq) provide a high number of polymorphic loci at a relatively low cost. A few GBS approaches were reported recently for sunflower [39,40,41,42], with all of them being based on the Elshire et al. [43] GBS protocol. Here, we present the first application of a ddRADseq approach for this crop, which involves the digestion of the genome using two different enzymes. The selection of the best enzyme pair is critical for assay success. The technique usually involves using one rare cutter (i.e., 6 bp recognition site) and one frequent cutter enzyme (i.e., 4 bp recognition site, [16]). However, given the size and complexity of the sunflower genome (3.6 Gb), two rare cutters (SphI and EcoRI) were used in this work, in order to obtain a significant reduction of genome complexity. Since repetitive regions account for more than 85% of the sunflower genome [12], a methylation-sensitive enzyme was included. A drawback of this genotyping technique is that the resulting data matrices often present high percentages of missing data. Indeed, almost a third of the SNPs identified before filtering had to be discarded due to a large number of missing genotypes (>80%). Imputation strategies are becoming an essential tool to overcome these limitations. Machine learning algorithms, such as the random forests (RF), are attractive approaches for imputing missing data, especially in large scale data sets for non-model organisms. Particularly, RF can deal with correlation and interaction among variables, while it generates an unbiased generalization error estimate, the out-of-bag error (OOB) [44]. In this work, the use of an imputation method based on RF and selection of predictors based on correlations between SNPs [26] allowed the reliable generation of complete SNP-matrices, with un unbiased OOB error fixed at 0.2.

The genotyping strategy implemented here combined proprietary ddRADseq with public WGS data to obtain an integrative SNP-matrix, including individuals from different breeding programs. This strategy allows not only to augment the number of individuals/populations under study but also to give significance to the big amount of publicly available NGS data, contributing to open data science and better use of the available resources. Although this is a powerful tool for both population and evolutionary genomics, there is no clear consensus on how to perform these analyses. Moreover, the use of a SNP-list to call variants in specific positions, instead of doing whole genome variant calling, reduces the computer processing time significantly, with mapping against the reference genome being the most computationally demanding step of the methodology. On the other hand, the main drawback of this combined genotyping strategy is that it restricts the queried SNPs to those polymorphic in the firstly genotyped dataset (i.e., INTA). However, this did not seem to impact significantly in our results, where each population showed genetic diversity levels similar to those reported in previous work (e.g., Mandel et al. [9]). In the present work, this approach allowed the generation of an 11,834 SNP-matrix, including the pre-breeding collections of INTA, INRA and USDA.

An initial characterization of the SNP-matrix showed that the markers are uniformly distributed across sunflower chromosomes, being the number of SNPs in accordance with chromosome length (i.e., a lower number of SNPs were called in the shorter chromosomes than in the larger chromosomes). This validates the performance of the ddRADseq assay, which is expected to generate evenly distributed markers across the genome [16]. Moreover, the prediction of variant effects showed that most of the markers fall within intergenic regions, and thus are likely to be neutral, confirming their usefulness for population genomics studies.

In general, the genetic diversity estimates obtained within the global and the individual breeding collections were moderate, with comparable levels of expected heterozygosity, independently of the population size. As expected when working with inbred lines, the observed heterozygosity values were low in each breeding collection. Differences in allele frequencies between breeding collections were apparent, not only when observing the minor allele frequency values but also when inspecting the allele frequency plots. The He values obtained here (~0.452) are higher than those reported by Mandel et al. [9] using a 10K Illumina SNP chip on the same USDA-UBC accessions (~0.404), suggesting that our ddRADseq method provides enough informative markers to conduct population studies in sunflower. In addition to generating a SNP panel with similar power to that of the chip, our ddRADseq strategy also allows new marker discovery avoiding ascertainment bias in new germplasm [20].

Our analysis of population structure revealed that differences among breeding programs explained only a small proportion of total genetic variation. However, although none of the clustering methods used here showed a direct correspondence with the origin of accessions, some groups were consistently recovered. This is the case for STRUCTURE Groups 6 and 3, which are composed of USDA-UBC accessions, and STRUCTURE Group 4, which mainly consists of INTA inbred lines. These distinct groups of accessions are of particular interest when planning the incorporation of new genetic backgrounds to each breeding collection. Among them, STRUCTURE group 4 contains a mixture of Argentinean HA and RHA inbred lines, bred for traits of agronomic importance such as drought stress tolerance and rust and sclerotinia head rot resistance [4,5,45].

Previous population studies based on these sunflower collections (i.e., INTA, Filippi et al. [5], USDA-UBC, Mandel et al. [8,9], INRA, Cadic et al. [11]) reported the maintainer/restorer status as the most prevalent characteristic associated with group delimitation. Here, the PCA constructed using the full panel of accessions (i.e., INTA, INRA and USDA-UBC) also showed a distinction between HA and RHA, along PC2. However, the low percentage of the variance captured by each of the first two PCs (7.84% and 6.98%, respectively), added to the variable number of subpopulations obtained using different clustering methods (K = 12, DAPC; K = 6, STRUCTURE; K = 9, distance-based methods), suggest that, when materials belonging to different breeding collections are pooled together, the imprint of each breeding program also becomes a key feature for cluster definition.

None of the groups were composed of INRA accessions only. This could be due to the lower representation of INRA accessions in our sample, together with the bias towards the variable sites identified in the INTA dataset, or to the fact that many of the accessions included in this collection were shared by the other two. The maintenance of genetic resources is essential for research and breeding purposes, but it is not a simple task. Some studies reported contamination, loss of genetic variability, genetic drift, among other constraints, occurring during the maintenance process in large germplasm collections [46,47]. In our work, we performed a comparative characterization based on the percentage of shared alleles of public inbred lines present in more than one breeding collection. Our results showed that none of the shared inbred lines were 100% identical among collections, but differentiation was below 10% for eight out of ten, with all clustering methods grouping them together. The remaining two, RHA299 and HAR2, had more than 18% differences. On one hand, RHA299 is a public inbred line originated in USDA and incorporated in INTA breeding programs years ago, so that percentage of differentiation could indicate contamination. On the other hand, HAR2 is a composite population (CP) derived from the variety Impira INTA (EEA Manfredi, [4]). The associated inbred line HAR2 registered at the USDA, Fargo, ND [48], was developed from this CP and is currently used as the international differential line for *Puccinia helianthi* [49]. So in that particular case, the differences observed between HAR2 could be due to the selection process performed in the different countries from the original CP. Nevertheless, the occurrence of these cases reinforces the idea of the need for monitoring breeding collections using not only phenotypic descriptors of variability but also genotypic descriptors [50].

Knowledge of linkage disequilibrium patterns can also help to the efficient use of breeding resources. This work presents whole genome linkage disequilibrium (LD) estimates as a function of a physical distance (bp) in sunflower. Whole genome LD estimates reported until now were based on genetic distance (i.e., cM, [9,11,51]), while using almost half of the molecular markers evaluated here (~5500 SNPs vs. ~11,800 SNPs). Overall patterns of LD decay show chromosome-specific behavior, which is generally consistent across breeding programs.

Chr10 showed the highest LD values, followed by Chr17, Chr5 and Chr2. Moreover, specific intra population LD patterns were observed, as high LD in Chr15, in INRA accessions. Mandel et al. [9] and Nambeesan et al. [51], who worked on the same USDA-UBC accessions, reported elevated LD in specific chromosomal regions, including portions of LGs 1, 5, 8, 10 and 13, while Cadic et al. [11], who worked on INRA accessions, reported high LD in LGs 5, 8, 10, 12, 14 and 17. Differences in distance estimates (i.e, bp vs. cM) preclude direct comparisons. However, our results of LD decay between and within breeding collections agree with those previous works on Chr10 having the highest LD, followed by Chr5 and Chr17. Visual inspection of the heatmap of pairwise LD values shows that both processes, reduced recombination and specific non-recombining regions, govern the high LD values observed in different regions of the sunflower genome. Mandel et al. [9] proposed that selection on plant architecture during sunflower domestication has shaped patterns of genetic diversity across the sunflower genome, with an important impact in Chr 10. In this regard, Owens et al. [52] showed that the extended LD on Chr10 could be a product of the wild introgression present in the fertility restoring male lines. On their work, Todesco et al. [53] reported a large, non-recombining haplotype block in Chr 5 containing two large inversions. According to these authors, inversions have been shown to control adaptive phenotypic variation (e.g., migration, color, flowering time), and to be associated with environmental clines [53]. It is important to mention that the extent of LD is population-specific and can be influenced by many factors, such as recombination and selection [54]. However, the conserved LD patterns observed among the collections examined here could be indicative of common aspects that had occurred during the selection process throughout the history of sunflower breeding

## 5. Conclusions

This work summarizes the most comprehensive characterization of sunflower genetic diversity encompassing breeding collections from INTA, INRA and USDA-UBC. Even though genetic differences were detected between breeding origins, they only explain 4.58% of the total variability. This fact added to the moderate genetic diversity estimates obtained here and similarities between LD patterns, suggest some homogeneity among international breeding materials, and a narrow genetic base of current sunflower breeding. In this regard, gene banks and crop wild relatives collections hold a substantial amount of genetic diversity for many agronomically important traits that can be exploited in order to expand the breeding genetic base and to cope with the changing environmental challenges for the crop.

## Figures and Tables

**Table 1:** Basic genetic diversity estimates within breeding collections.

	*n*	He			Ho	% of SNPs with MAF <0.05
		Min	Mean	Max		
**INTA**	135	0.022	0.454	0.500	0.007	-
**INRA**	58	0.000	0.452	0.500	0.013	0.072
**USDA-UBC**	289	0.003	0.454	0.500	0.030	0.005
**3 POPULATIONS**	482	0.019	0.454	0.500	0.022	0.053

**Table 2:** Percentage of identity between public sunflower inbred lines present in more than one breeding collection.

Accession Name	Code 1 (USDA-UBC/INRA)	Code 2 (INTA)	% of Identity
**HA853**	SAM002 (USDA)	PMA102 (INTA)	0.92
**RHA299**	SAM169 (USDA)	PMA55 (INTA)	0.82
**HA64**	SAM172 (USDA)	PMA124 (INTA)	0.93
**HA89**	SAM173 (USDA)	PMA78 (INTA)	0.97
**HA234**	SAM176 (USDA)	PMA80 (INTA)	0.92
**HAR2**	SAM227 (USDA)	PMA97 (INTA)	0.78
**RHA266**	SF268 (INRA)	PMA133 (INTA)	0.94
**PAC2**	SF302 (INRA)	PMA132 (INTA)	0.92
**RHA801**	SF330 (INRA)	PMA119 (INTA)	0.92
**RHA274**	SF332 (INRA)	PMA123 (INTA)	0.93

## Supplementary Materials

The following are available online at https://www.mdpi.com/2073-4425/11/3/283/s1. Figure S1: Transition and transversion counts for the 34488 SNPs identified in the INTA accessions (*n* = 135) through ddRADseq. Figure S2: Individual heterozygosis plots (represented as number of copies of the reference alleles), between and within breeding collections. (A) INTA (*n* = 135); (B) INRA (*n* = 58); (C) USDA-UBC (*n* = 489); (D) The full panel of accessions (*n* = 482). Figure S3: Results of STRUCTURE for K = 6. A. Population structure in the full panel of accessions (*n* = 482) assessed with 11,834 SNPs. Figure S4: Identity-by-descent dendrogram for the full panel of accessions (*n* = 482) assessed with 11,834 SNPs. Figure S5: Scatter plot from a Principal Component Analysis for the full panel of accessions (*n* = 482) assessed with 11,834 SNPs. Accessions were colored according to their maintainer (HA)/restorer (RHA) status. Accessions for which no information was available were classified as N/A. Figure S6: Linkage disequilibrium (r^2^) vs distance (bp) for the INTA accessions (*n* = 135). A cut-off line was plotted at r^2^ = 0.2. The blue line represents the y ~log(x) function. (A)–(Q) Sunflower chromosomes 1 to 17. CHR = Chromosome. Figure S7: Linkage disequilibrium (r^2^) vs distance (bps) for the INRA accessions (*n* = 58). A cut-off line was plotted at r^2^ = 0.2. The blue line represents the y ~log(x) function. (A)–(Q) Sunflower chromosomes 1 to 17. CHR = Chromosome. Figure S8: Linkage disequilibrium (r^2^) vs distance (bp) for the USDA-UBC accessions (*n* = 289). A cut-off line was plotted at r^2^ = 0.2. The blue line represents the y ~log(x) function. (A)–(Q) Sunflower chromosomes 1 to 17. CHR = Chromosome. Figure S9: Heat map of linkage disequilibrium for the full panel of accessions, per chromosome. Individual data points reflect pairwise LD values between markers. Note that the values above and below the diagonal are identical. CHR = Chromosome. File S1: Command line used for variant calling from whole genome sequencing data. Table S1: Sunflower accessions included in this study, breeding collection of origin, group assignment according to DAPC and STRUCTURE, and the relatedness analysis using Identity-By-Descent (IBD) measures. Table S2: Number of INTA/INRA/USDA-UBC accessions in each inferred group. (A) DAPC; (B) STRUCTURE; (C) Relatedness analysis using Identity-By-Descent (IBD) measures. Table S3: Percentage of accessions assigned to each group using DAPC or STRUCTURE clustering methods that were clustered together in the dendrogram. (A) DAPC; (B) STRUCTURE. Table S4: Linkage disequilibrium statistics between and within breeding collections.
