# Supplementary material for: Genetic Diversity, Population Structure and Linkage Disequilibrium Assessment among International Sunflower Breeding Collections
Source: Genes (Basel). 2020 Mar 6;11(3):283. doi: 10.3390/genes11030283 (PMC7140877; doi:10.3390/genes11030283)
Supplement: Supplementary file 1 [file genes-11-00283-s001.zip › Supplementary_Filippi_et_al/File_S1.docx]

## Introduction

This file resumes the command line used for variant calling in sunflower Illumina whole genome sequencing data (article: “Genetic diversity, population structure and linkage disequilibrium assessment among international sunflower breeding collections”). It covers data quality checking using FASTQC, read trimming using TRIMMOMATIC, alignment against the reference using BOWTIE2, data preparation and variant calling (Single Nucleotide Polymorphisms, SNPs) using GATK.

### Steps:

#### a) Data download:

Sunflower public data was downloaded from ENA (European Nucleotide Archive), projects number PRJNA353001 and SRP092899XXX) using:

wget -c link_to_the_ENA_projects

#### b) Data quality inspection, using FASTQC. Identification of possible adapter sequences:

mkdir log; ls -1 *_paired.fq.gz | xargs -n 1 -I XXX -- bsub -J XXX_fastqc -q 'production-rh74' -M 1000 -R 'rusage[mem=1000]' -n 4 -o log/XXX.stdout -e log/XXX.stderr '/PATH/lib/FastQC/fastqc XXX'

#### c) Run Trimmomatic in order to discard low quality reads, discard very short reads (i.e. < 50 bp) and remove adapters.

The adapter sequences were retrieved from Illumina webpage. Given that both Truseq and Nextera adapters were present in the raw data, both adapter pairs were used for clipping. The adapter file was created as following:

nano TruSeq3Next-PE.fa

>Trans2_rc

CTGTCTCTTATACACATCTGACGCTGCCGACGA

>Trans1_rc

CTGTCTCTTATACACATCTCCGAGCCCACGAGAC

>PE1_rc

AGATCGGAAGAGCACACGTCTGAACTCCAGTCA

>PE2_rc

AGATCGGAAGAGCGTCGTGTAGGGAAAGAGTGT

The command line used was:

mkdir -p log_trimming_adap

mkdir trim_adap

TRIMMER='java -jar /PATH/Trimmomatic-0.39/trimmomatic-0.39.jar PE -phred33'

TRIMMER_OPTS='ILLUMINACLIP:TruSeq3Next-PE.fa:2:30:10 LEADING:3 TRAILING:3 SLIDINGWINDOW:4:15 MINLEN:50'

ls *_{1,2}.fastq.gz| cut -f 1 -d '_' | sort | uniq |

xargs -I XXX -n 1 -- bsub -J XXX_trimming -q 'research-rh74' -M 2000 -R 'rusage[mem=2000]' -n 4 -o log_trimming_adap/XXX.stdout -e log_trimming_adap/XXX.stderr "$TRIMMER XXX_1.fastq.gz XXX_2.fastq.gz trim_adap/XXX_1_paired.fq.gz trim_adap/XXX_1_unpaired.fq.gz trim_adap/XXX_2_paired.fq.gz trim_adap/XXX_2_unpaired.fq.gz $TRIMMER_OPTS"

#### d) Map the reads to the reference, using Bowtie2:

i. Index the genome

bowtie2-build GCF_002127325.1_HanXRQr1.0.fa GCF_002127325.1_HanXRQr1.0

ii. Map the reads against the reference (*default* parameters; the mapping quality will be filtered when doing the variant calling):

mkdir -p log_align

mkdir -p unmapped

ALIGNER='bowtie2'

ALIGNER_OPTS='-x /PATH/bowtie2/helianthusXRQ1.0 -t --un unmapped/XXX.single.un --un-conc unmapped/XXX.pair.un -S XXX.sam'

ls *_{1_paired,1_unpaired,2_paired,2_unpaired}.fq.gz| cut -f 1 -d '_' | sort | uniq |

xargs -I XXX -n 1 -- bsub -J XXX_align -q 'production-rh74' -M 4000 -R 'rusage[mem=4000]' -n 4 -o log_align/XXX.stdout -e log_align/XXX.stderr "$ALIGNER -1 XXX_1_paired.fq.gz -2 XXX_2_paired.fq.gz -U XXX_1_unpaired.fq.gz,XXX_2_unpaired.fq.gz $ALIGNER_OPTS"

#### e) Generate BAM files from SAM files, using Samtools:

mkdir bam

mkdir logbam

SAMBAM='samtools view'

SAMBAM_OPTS='-t 8 -o bam/XXX.bam'

ls -1 *.sam |

ls *.sam| cut -f 1 -d '.' | sort |

xargs -n 1 -I XXX -- bsub -J XXX.bam -q 'production-rh74' -M 2000 -R 'rusage[mem=2000]' -n 4 -o logbam/XXX.stdout -e logbam/XXX.stderr "$SAMBAM -b XXX.sam $SAMBAM_OPTS"

#### f) Sort the BAM by coordinates, using Samtools:

mkdir sortedbam

mkdir logsortbam

SORTBAM='samtools sort'

SORTBAM_OPTS='-t 8 -o sortedbam/XXX_sorted.bam'

ls *.bam| cut -f 1 -d '.' | sort |

xargs -n 1 -I XXX -- bsub -J XXX_sorted.bam -q 'production-rh74' -M 2000 -R 'rusage[mem=2000]' -n 4 -o logsortbam/XXX.stdout -e logsortbam/XXX.stderr "$SORTBAM XXX.bam $SORTBAM_OPTS"

#### g) Add a read group to each sorted BAM file, using Picard (required by variant callers):

mkdir addRG

mkdir logaddRG

mkdir tmp

ADDRG='java -jar /PATH/lib/picard.jar AddOrReplaceReadGroups'

ADDRG_OPTS='RGID=XXX RGLB=XXX RGPL=illumina RGPU=run RGSM=XXX SORT_ORDER=coordinate CREATE_INDEX=TRUE VALIDATION_STRINGENCY=LENIENT O=addRG/XXX_RG.bam TMP_DIR=tmp'

ls *.bam| cut -f 1 -d '.' | sort |

xargs -n 1 -I XXX -- bsub -J XXX_RG -q 'research-rh74' -M 4000 -R 'rusage[mem=4000]' -n 4 -o logaddRG/XXX.stdout -e logaddRG/XXX.stderr "$ADDRG I=XXX.bam $ADDRG_OPTS"

#### h) Validate each BAM file, using Picard:

mkdir logvalbam

mkdir tmp

VALBAM='java -jar /PATH/lib/picard.jar ValidateSamFile'

VALBAM_OPTS='R=/PATH/bowtie2/GCF_002127325.1_HanXRQr1.0_genomic.fasta MODE=SUMMARY TMP_DIR=tmp IGNORE=INVALID_TAG_NM'

ls *.bam| cut -f 1 -d '.' | sort |

xargs -n 1 -I XXX -- bsub -J XXX_VAL -q 'production-rh74' -M 8000 -R 'rusage[mem=8000]' -n 4 -o logvalbam/XXX.stdout -e logvalbam/XXX.stderr "$VALBAM I=XXX.bam $VALBAM_OPTS"

#### i) Mark duplicates, using Picard:

mkdir MD

mkdir logMD

mkdir tmp2

MD='java -jar /PATH/lib/picard.jar MarkDuplicates'

MD_OPTS='M=XXX_metrics.txt O=MD/XXX_MD.bam TMP_DIR=tmp2'

ls *.bam| cut -f 1 -d '.' | sort |

xargs -n 1 -I XXX -- bsub -J XXX_MD -q 'production-rh74' -M 8000 -R 'rusage[mem=8000]' -n 4 -o logMD/XXX.stdout -e logMD/XXX.stderr "$MD I=XXX.bam $MD_OPTS"

#### j) Index the BAM file, using Samtools:

mkdir logIB

IB='samtools index'

ls *.bam| cut -f 1 -d '.' | sort |

xargs -n 1 -I XXX -- bsub -J XXX_MD -q 'production-rh74' -M 500 -R 'rusage[mem=500]' -n 4 -o logIB/XXX.stdout -e logIB/XXX.stderr "$IB XXX.bam"

#### k) SNP calling using GATK: genotyping a given set of positions.

#### Given that we were just interested in a list of SNPs, and not in whole genome *de novo* variant calling, we gave the positions to be queried in a VCF format as input (example):

```
Example of VCF with the positions to be queried:
##fileformat=VCFv4.0
#CHROM POS ID REF ALT QUAL FILTER INFO
NC_035433.1 134752 . A G . . .
NC_035433.1 134946 . T C . . .
NC_035433.1 392972 . C T . . .
NC_035433.1 392995 . C T . . .
NC_035433.1 443310 . G T . . .

The command used was:
mkdir GATK_GA
mkdir logGATK_GA
GA='java -jar /PATH/bin/gatk-3.5/GenomeAnalysisTK.jar'
GA_OPTS='-T UnifiedGenotyper -R ../PATH/genome/GCF_002127325.1_HanXRQr1.0.fa --alleles /PATH/vcf/PMA-INTA.vcf -L /PATH/vcf/PMA-INTA.vcf --genotyping_mode GENOTYPE_GIVEN_ALLELES -o GATK_GA_AS/XXX_snpsAS.vcf --min_base_quality_score 30 -stand_call_conf 30 '
ls *.bam| cut -f 1 -d '.' | sort |
xargs -n 1 -I XXX -- bsub -J XXX_GA_AS -q 'production-rh74' -M 8000 -R 'rusage[mem=8000]' -n 4 -o logGATK_GA/XXX.stdout -e logGATK_GA/XXX.stderr "$GA -I XXX.bam $GA_OPTS"
```

### References

- ANDREWS, Simon, et al. FastQC: a quality control tool for high throughput sequence data. 2010.
- BOLGER, Anthony M.; LOHSE, Marc; USADEL, Bjoern. Trimmomatic: a flexible trimmer for Illumina sequence data. *Bioinformatics*, 2014, vol. 30, no 15, p. 2114-2120.
- LANGMEAD, Ben; SALZBERG, Steven L. Fast gapped-read alignment with Bowtie 2. *Nature methods*, 2012, vol. 9, no 4, p. 357.
- LI, Heng, et al. The sequence alignment/map format and SAMtools. *Bioinformatics*, 2009, vol. 25, no 16, p. 2078-2079.
- MCKENNA, Aaron, et al. The Genome Analysis Toolkit: a MapReduce framework for analyzing next-generation DNA sequencing data. *Genome research*, 2010, vol. 20, no 9, p. 1297-1303.
